# Targeting the mRNA-binding protein HuR impairs malignant characteristics of pancreatic ductal adenocarcinoma cells

**DOI:** 10.18632/oncotarget.4743

**Published:** 2015-07-25

**Authors:** Masaya Jimbo, Fernando F. Blanco, Yu-Hung Huang, Aristeidis G. Telonis, Brad A. Screnci, Gabriela L. Cosma, Vitali Alexeev, Gregory E. Gonye, Charles J. Yeo, Janet A. Sawicki, Jordan M. Winter, Jonathan R. Brody

**Affiliations:** ^1^ Department of Surgery and The Jefferson Pancreas, Biliary and Related Cancer Center, Thomas Jefferson University, Philadelphia, PA, USA; ^2^ Department of Pharmacology & Experimental Therapeutics, Division of Clinical Pharmacology, Thomas Jefferson University, Philadelphia, PA, USA; ^3^ Department of Biochemistry and Molecular Biology, Drexel University College of Medicine, Philadelphia, PA, USA; ^4^ Computational Medicine Center, Sidney Kimmel Medical College, Thomas Jefferson University, Philadelphia, PA, USA; ^5^ Department of Microbiology and Immunology, Kimmel Cancer Center, Thomas Jefferson University, Philadelphia, PA, USA; ^6^ Department of Dermatology, Thomas Jefferson University, Philadelphia, PA, USA; ^7^ NanoString Technologies, Seattle, WA, USA; ^8^ Lankenau Institute for Medical Research, Wynnewood, PA, USA

**Keywords:** pancreatic ductal adenocarcinoma, pancreatic cancer, post-transcriptional regulation, HuR, ELAVL1

## Abstract

Post-transcriptional regulation is a powerful mediator of gene expression, and can rapidly alter the expression of numerous transcripts involved in tumorigenesis. We have previously shown that the mRNA-binding protein HuR (*ELAVL1*) is elevated in human pancreatic ductal adenocarcinoma (PDA) specimens compared to normal pancreatic tissues, and its cytoplasmic localization is associated with increased tumor stage. To gain a better insight into HuR’s role in PDA biology and to assess it as a candidate therapeutic target, we altered HuR expression in PDA cell lines and characterized the resulting phenotype in preclinical models. HuR silencing by short hairpin and small interfering RNAs significantly decreased cell proliferation and anchorage-independent growth, as well as impaired migration and invasion. In comparison, HuR overexpression increased migration and invasion, but had no significant effects on cell proliferation and anchorage-independent growth. Importantly, two distinct targeted approaches to HuR silencing showed marked impairment in tumor growth in mouse xenografts. NanoString nCounter^®^ analyses demonstrated that HuR regulates core biological processes, highlighting that HuR inhibition likely thwarts PDA viability through post-transcriptional regulation of diverse signaling pathways (e.g. cell cycle, apoptosis, DNA repair). Taken together, our study suggests that targeted inhibition of HuR may be a novel, promising approach to the treatment of PDA.

## INTRODUCTION

Pancreatic ductal adenocarcinoma (PDA) is currently the fourth most common cause of cancer-related death in the United States, yet will likely become the second leading cause of cancer-related death by 2020, behind only non-small cell lung cancer [1]. The development of effective therapies achieved for other common cancers (e.g. breast, prostate, colorectal) has so far eluded PDA, despite the vastly improved understanding of underlying genetic alterations (e.g. *KRAS*, *p16/CDKN2A*, *TP53*) and dysregulated signaling pathways involved in pancreatic tumorigenesis [2–5]. In fact, since the introduction in 1997 of gemcitabine (2′,2′-difluorodeoxycytidine) monotherapy as the standard of care for metastatic PDA, the only new treatment regimens to show superior efficacy (gemcitabine + erlotinib, gemcitabine + Abraxane, and FOLFIRINOX) have improved overall survival in the metastatic setting by only 0.4–4.3 months [2, 6]. New approaches in the treatment of this deadly disease are urgently needed [7].

Genetic mutations and copy number changes can dramatically influence gene expression, but they emerge in cancer cells over many years of biologic selection [8]. Molecular pathway changes at the RNA level represent a separate, but understudied, aspect of cancer biology that is especially relevant for adaptive cellular reprogramming to acute stress [9]. RNA expression changes are rapid, efficient, and reversible [9–11]. Broadly speaking, these changes may be classified as *transcriptional* and impact the quantity of RNA made by a cancer cell, or *post-transcriptional* and affect other aspects of RNA regulation (e.g. stability, translation). Post-transcriptional regulation is predominantly mediated by *trans*-acting microRNAs (miRNAs) and RNA-binding proteins (RBPs), many of which have become implicated in cancer progression [12–15]. While miRNAs have received greater attention in recent years, RBPs may be even more important for the tumorigenesis process [16–18]. RBPs are more stable and require a greater investment of cell energy for synthesis; moreover, roughly 5% of all genes are believed to encode RBPs [19].

Human antigen R (HuR, encoded by the *ELAVL1* gene) is a ubiquitously expressed RBP whose role in cancer has become increasingly evident in recent years [20]. HuR is primarily localized to the nucleus, where it is involved in pre-messenger RNA (mRNA) processing, but can shuttle to the cytoplasm where it regulates the stability and/or translation of bound mRNA transcripts [21]. HuR targets have characteristic AU-rich RNA elements (AREs), which are typically located in the 3′ untranslated region (3′ UTR). Many of these transcripts are involved in key cellular processes such as proliferation, survival, angiogenesis, immune response, and metastasis, enabling HuR to influence multiple critical survival mechanisms [20–22].

No somatic *ELAVL1* mutations, copy number changes, or epigenetic alterations in any human cancer have been reported to date [23, 24]. Yet clinically, we and others have demonstrated that total and/or cytoplasmic HuR expression is elevated in numerous tissue-specific cancers, compared to normal cells [23, 25–30]. In general, elevated HuR expression and/or localization in the cytoplasm (where HuR carries out the majority of its mRNA-regulating functions) are associated with poor clinicopathologic features, suggesting that HuR is a potent promoter of tumorigenesis or aggressive cancer biology [23, 25–29, 31–52]. Specifically in PDA, we have shown that HuR expression (both total and cytoplasmic) is elevated compared to normal pancreatic tissues, and that cytoplasmic HuR expression positively correlates with tumor (T) stage [25, 30]. We have also demonstrated using *in vitro* models that HuR protects PDA cells from stressors that are relevant to the tumor microenvironment, such as glucose withdrawal, hypoxia, and DNA damage (Blanco et al., unpublished) [53, 54]. These stressors act as stimuli to translocate HuR to the cytoplasm, wherein it stabilizes and promotes the translation of target mRNA transcripts (e.g. mediators of glucose metabolism, the hypoxia-inducible proto-oncogene *PIM1*, the mitotic kinase inhibitor *WEE1*) in a manner that promotes cell survival.

The differential expression of HuR between neoplastic and normal tissues (i.e. a more available target in cancer cells vs. normal cells), combined with HuR’s induction of numerous pro-tumorigenic transcripts over multiple defined PDA core signaling pathways, supports the hypothesis that HuR is a promising, novel therapeutic target in PDA [3, 20]. In fact, several publications have explored the effect of modulating HuR expression in various tissue-specific cancers (e.g. breast, colorectal, brain) [23, 55–57]. In the majority of cases, overexpression of HuR enhances tumor proliferation, whereas silencing of HuR reduces tumor proliferation. Therefore, we launched into a line of investigation utilizing preclinical models to test the hypothesis that HuR drives aggressive PDA biology, and may be targeted as a novel treatment strategy for PDA.

## RESULTS

### Characterization of doxycycline (DOX)-inducible MIA PaCa-2 cell lines

**Figure 1:**
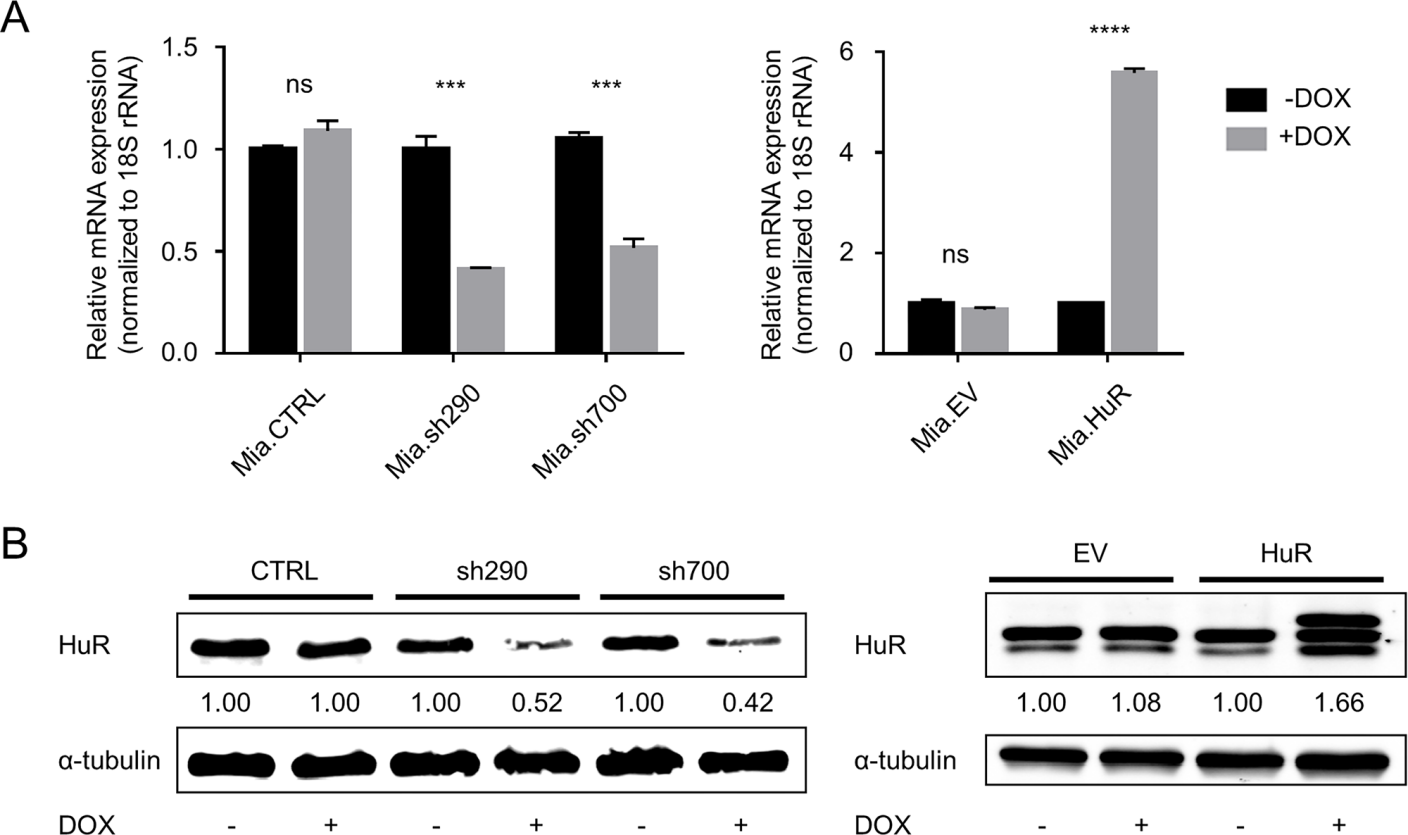
Characterization of DOX-inducible MIA PaCa-2 cell lines **A.** qPCR analysis of HuR mRNA expression in Mia.CTRL, Mia.sh290, and Mia.sh700 cells treated with 0 or 2 μg/ml DOX for 5 days (*left*), and Mia.EV and Mia.HuR cells treated with 0 or 2 μg/ml DOX for 2 days (*right*), normalized to 18S rRNA expression. Mia.sh290 and Mia.sh700 are inducible HuR-silencing cell lines, whereas Mia.HuR is an inducible HuR-overexpressing cell line. Mia.CTRL and Mia.EV are the respective control cell lines. **B.** Western blotting analysis of HuR protein expression in Mia.CTRL, Mia.sh290, and Mia.sh700 cells treated with 0 or 2 μg/ml DOX for 5 days (*left*), and Mia.EV and Mia.HuR cells treated with 0 or 2 μg/ml DOX for 2 days (*right*), normalized to alpha-tubulin protein expression. *ns* = non-significant; *** = *p* < 0.001; **** = *p* < 0.0001.

In order to study the effect of HuR expression on PDA phenotype, we generated DOX-inducible MIA PaCa-2 cell lines. Two cell lines generated by lentiviral transduction express distinct short hairpin RNAs (shRNAs) that target HuR mRNA, in response to DOX treatment (hereafter referred to as Mia.sh290 and Mia.sh700, based on the locations of targeted sequences). In DOX-treated Mia.sh290 and Mia.sh700 cells, we observed significant knockdown of HuR at both mRNA and protein levels. The mRNA knockdown in response to DOX was 59% and 48% in Mia.sh290 and Mia.sh700, respectively (*p* < 0.001) (Fig. 1A). The protein knockdown reached a maximum of 50–60% in both cell lines at 5 days of DOX treatment, and was sustained thereafter (Figs. 1B and S1). HuR expression in a control cell line, stably transduced with empty vector lentivirus (hereafter referred to as Mia.CTRL), was unaffected by DOX treatment.

Another cell line (hereafter referred to as Mia.HuR) was generated by stable transfection with a tetracycline-responsive plasmid, and overexpressed HuR in response to DOX treatment, with 5.6-fold overexpression at the mRNA level (*p* < 0.0001) and 1.5–2-fold overexpression at the protein level (Fig. 1). HuR expression in a control cell line, stably transfected with empty vector (hereafter referred to as Mia.EV), was unaffected by DOX treatment.

### HuR is required for short-term proliferation of PDA cells

**Figure 2:**
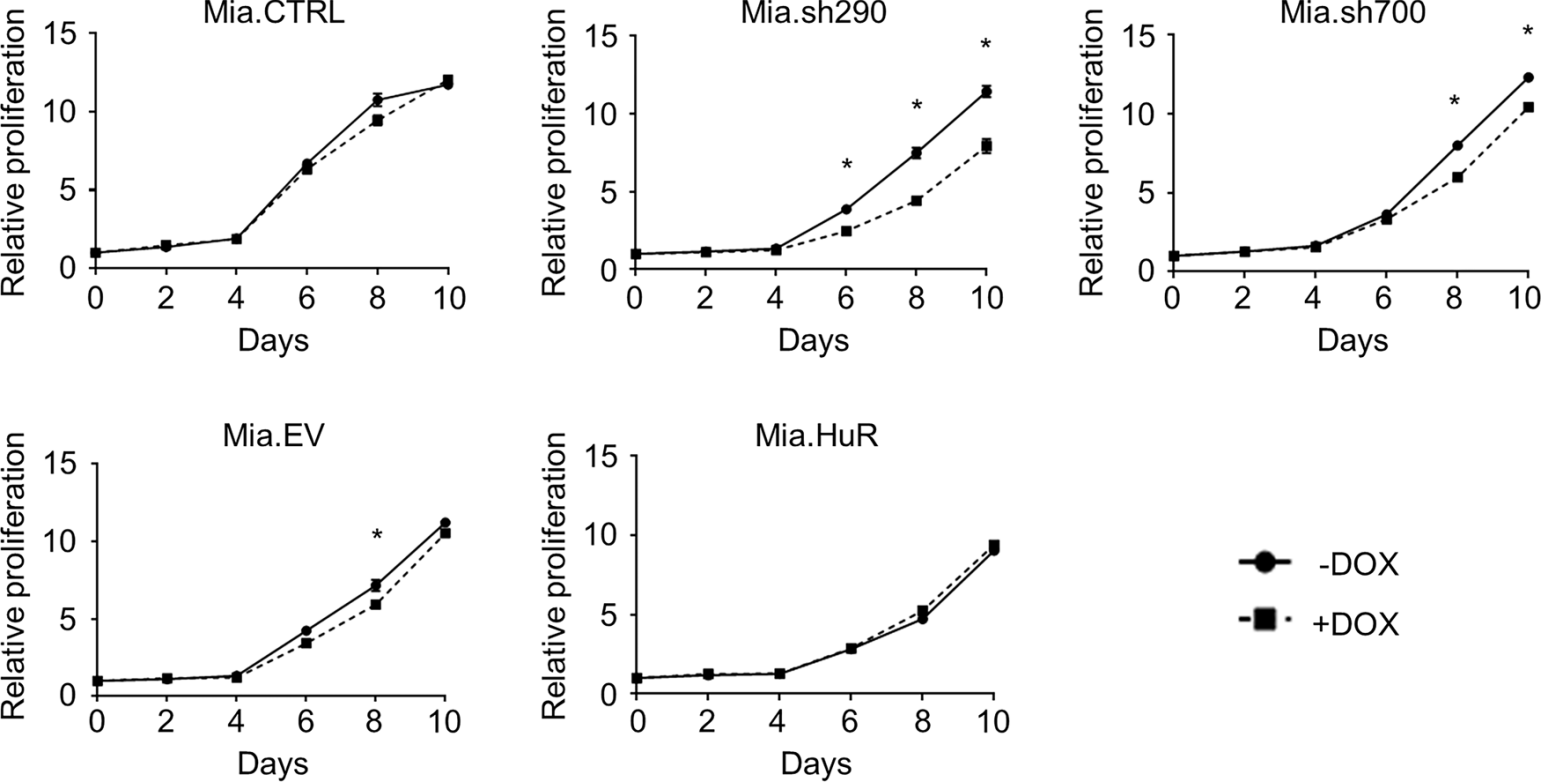
HuR is required for short-term proliferation of PDA cells Relative proliferation of DOX-inducible MIA PaCa-2 cell lines treated with 0 or 2 μg/ml DOX for the indicated time points, as determined by measurement of dsDNA content by PicoGreen staining. Each data point represents the mean of 5 independent experiments ± standard error of the mean (SEM). * = *p* < 0.05.

We first studied the effect of manipulating HuR expression on cell proliferation. DOX treatment caused a significant decrease in the proliferation of Mia.sh290 and Mia.sh700 cells over a 10-day period, as assessed by PicoGreen staining of double-stranded DNA (dsDNA) content (Fig. 2). The decrease did not become apparent until 5–6 days of treatment, likely due to the fact that DOX-induced HuR silencing is gradual and does not reach maximal protein-level knockdown until 4–5 days of treatment (Fig. S1). To confirm that the effect of HuR manipulation was not cell line-specific, we performed transient transfections in an additional PDA cell line (PL5). In contrast to the gradual effect of DOX treatment in Mia.sh290 and Mia.sh700 cells, rapid HuR silencing in PL5 cells by small interfering RNA (siRNA) transfection resulted in immediate and potent suppression of cell proliferation (Fig. S2). Surprisingly, HuR overexpression had no apparent effect on cell proliferation, in both the DOX-treated Mia.HuR cells and PL5 cells transiently transfected with HuR overexpression plasmid (Figs. 2 and S2).

### HuR is required for anchorage-independent growth of PDA cells

**Figure 3:**
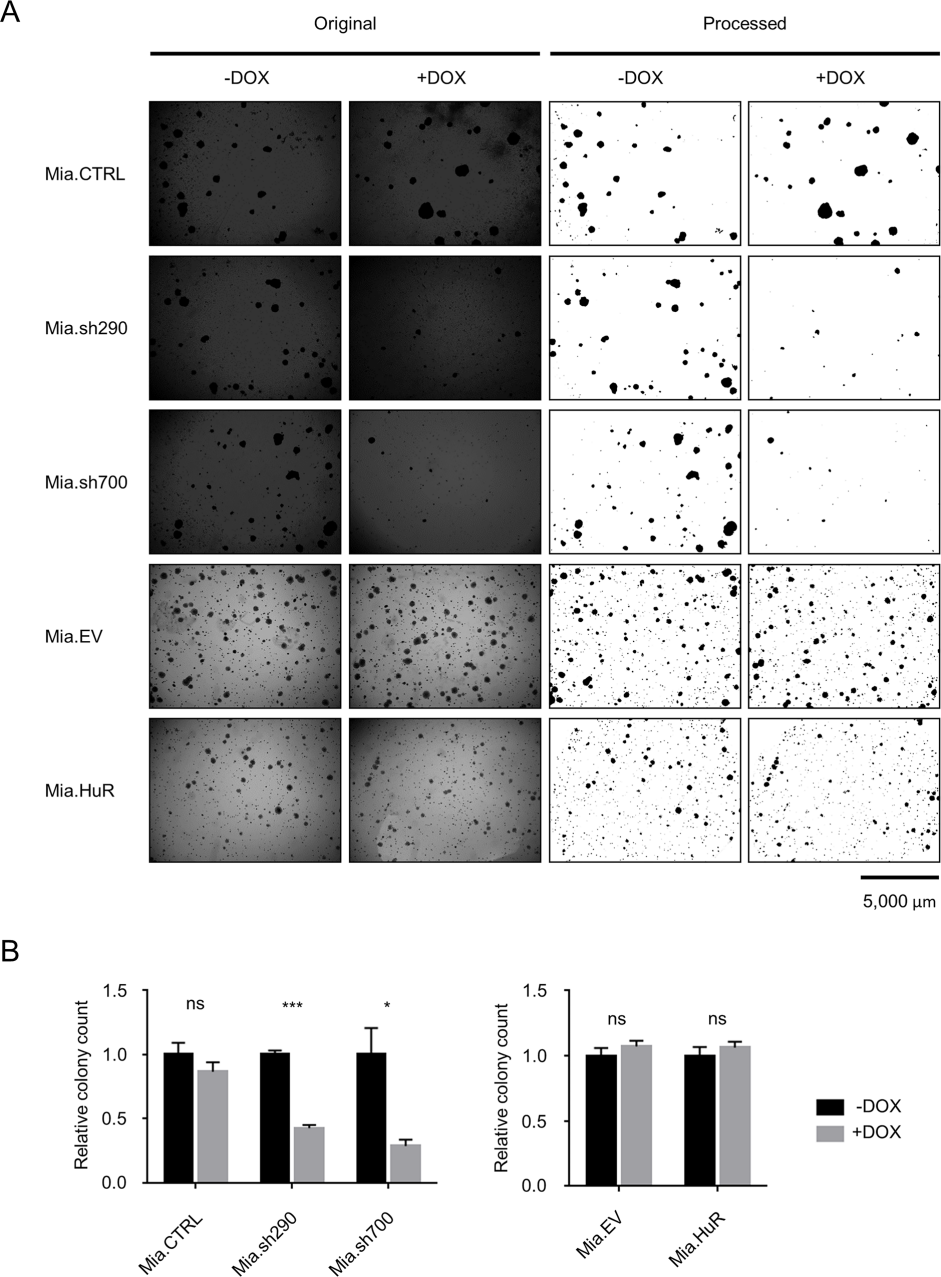
HuR is required for anchorage-independent growth of PDA cells **A.** Representative images of crystal violet-stained colonies of DOX-inducible MIA PaCa-2 cell lines cultured in 0 or 2 μg/ml DOX for 4 weeks. Left side shows the original images. Right side shows the images processed in ImageJ for clarity and colony counts. **B.** Relative quantification of colony counts. Each bar represents the mean of 3 independent experiments ± SEM. *ns* = non-significant, * = *p* < 0.05; *** = *p* < 0.001.

There was a possibility that the full effect of manipulating HuR expression on PDA proliferation could not be appreciated in the short timescale of the above experiment. As such, we performed soft agar colony formation assays with the DOX-inducible MIA PaCa-2 cell lines to gauge anchorage-independent growth over a 4 week period (Fig. 3). Cells were seeded in soft agar, and cultured in the presence or absence of DOX for 4 weeks. In the Mia.sh290 and Mia.sh700 cell lines, DOX-induced HuR silencing resulted in 57% and 71% decrease in colony number, compared to the untreated condition (*p* < 0.001 and *p* < 0.05, respectively). As with the short-term proliferation assay, DOX treatment had no effect on colony formation for the Mia.CTRL, Mia.EV, and Mia.HuR cell lines. Taken together, these results demonstrate that inhibition of endogenous HuR expression compromises the normal proliferation of PDA; however, further increases in HuR expression beyond endogenous levels do not enhance cell proliferation.

### HuR facilitates PDA invasiveness

**Figure 4:**
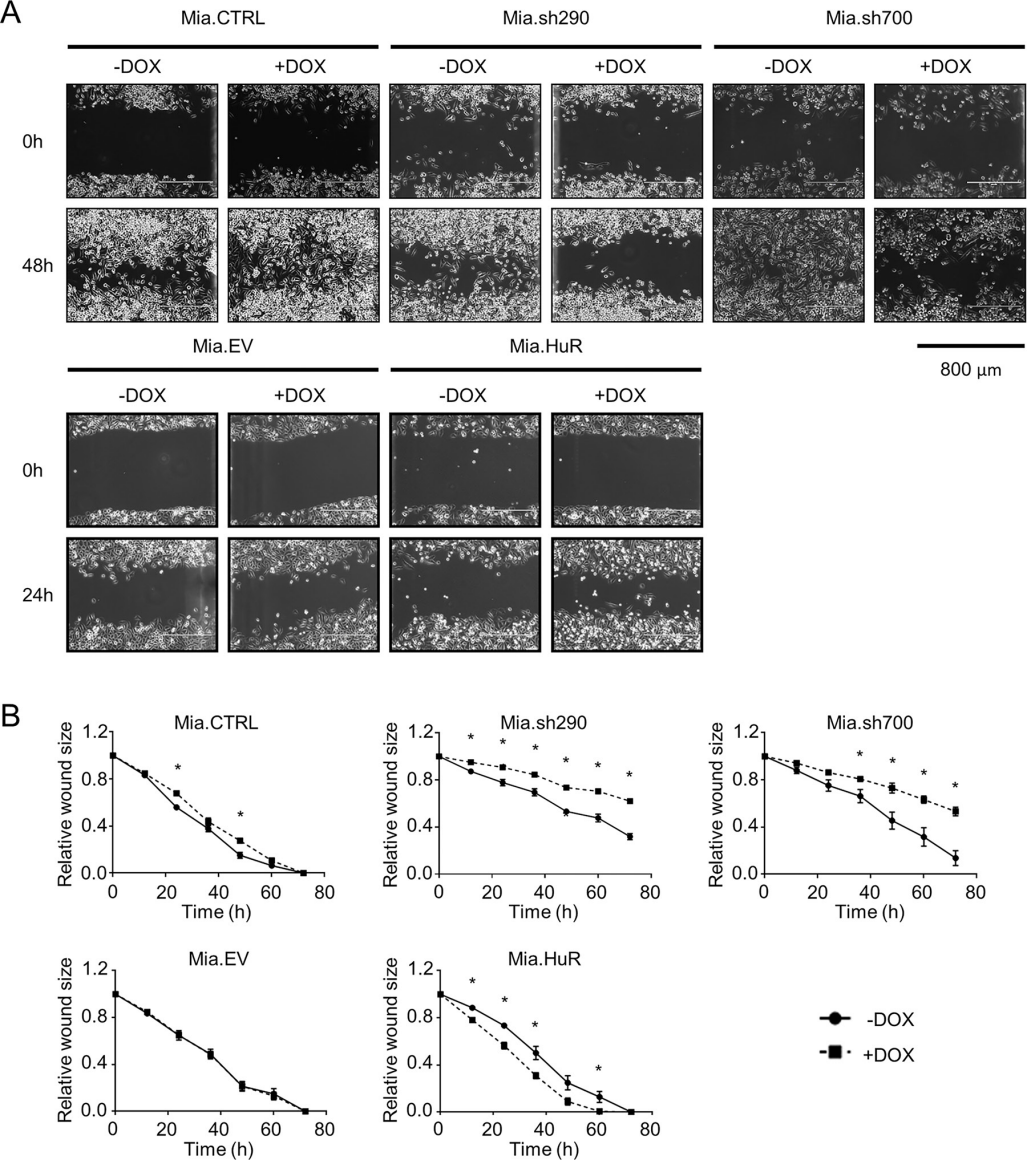
HuR promotes PDA migration **A.** Representative images of *in vitro* scratch assays performed with DOX-inducible MIA PaCa-2 cell lines pre-treated with 0 or 2 μg/ml DOX for 5 days. Images were taken at 0 h and 48 h post-scratch for the Mia.CTRL, Mia.sh290, and Mia.sh700 cell lines, and at 0 h and 24 h post-scratch for the Mia.EV and Mia.HuR cell lines. **B.** Quantification of the rate of scratch closure, as measured by change in wound size relative to the 0 h time point. Each data point represents the mean of 3 independent experiments ± SEM. * = *p* < 0.05.

We next investigated the importance of HuR expression on the invasive phenotype of PDA using two different assays. *In vitro* scratch assays with Mia.sh290 and Mia.sh700 cells pre-treated with 0 or 2 μg/ml DOX showed a significant decrease in migration rate under HuR-silenced conditions (*p* < 0.05) (Fig. 4). As expected, Mia.CTRL and Mia.EV cells were not significantly affected by DOX treatment. Whereas it did not affect proliferation of PDA cells, HuR overexpression by DOX treatment of Mia.HuR cells significantly increased the migration rate (*p* < 0.05).

**Figure 5:**
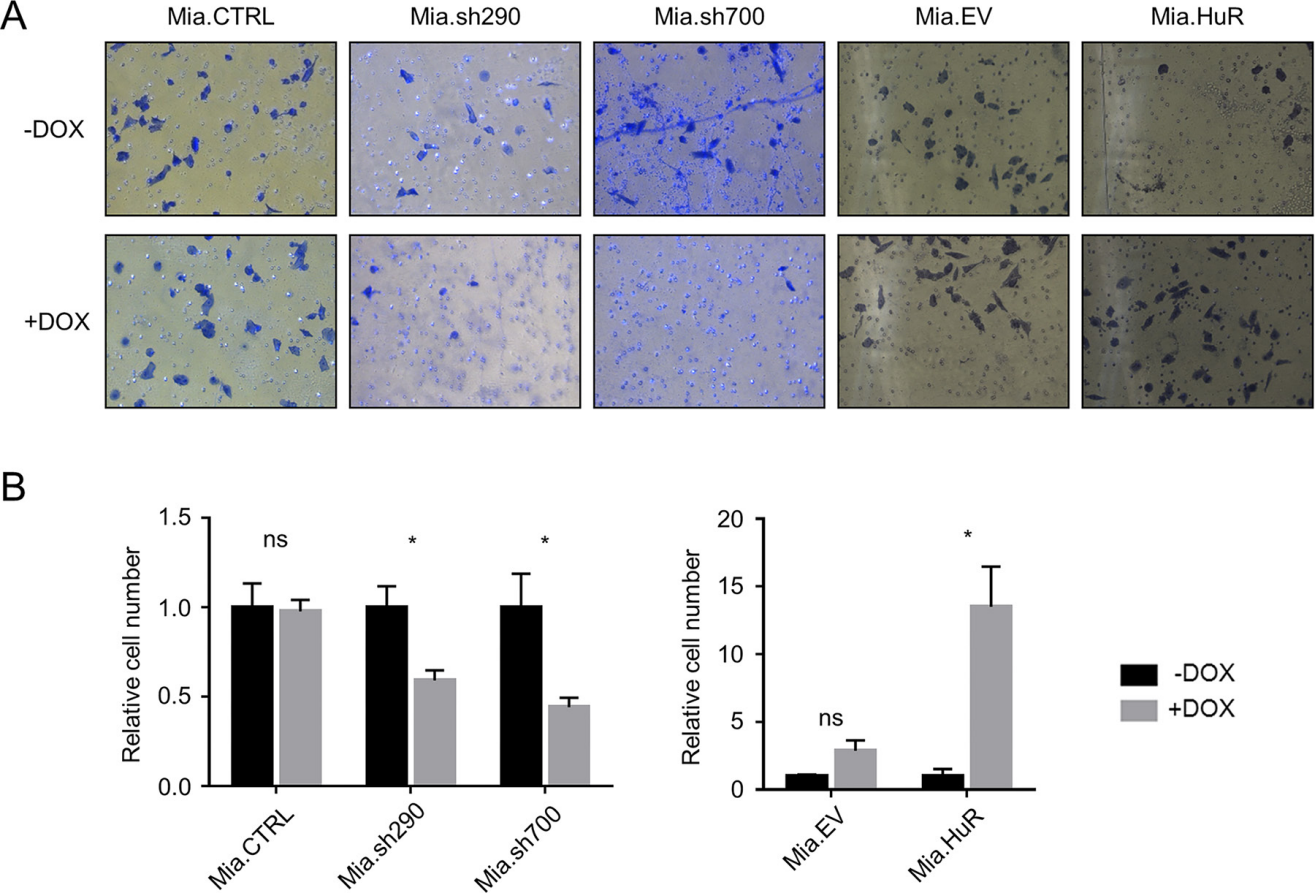
HuR promotes PDA invasion through an extracellular matrix analogue **A.** Representative images of Matrigel invasion assays performed with DOX-inducible MIA PaCa-2 cell lines pre-treated with 0 or 2 μg/ml DOX for 5 days. Cells that invaded through the Matrigel and onto the basal surface of transwell inserts were stained with Differential Quik and photographed at 20X magnification. **B.** Quantification of Matrigel invasion assays. Values for each cell line were normalized to the number of cells in the untreated condition. Each bar represents the mean of 3 independent experiments ± SEM. *ns* = non-significant; * = *p* < 0.05.

To complement the *in vitro* scratch assays, we performed Matrigel invasion assays, which showed the same trends (Fig. 5). Cells in serum-free medium were seeded on transwell inserts coated with Matrigel, and incubated for 24 hours with serum-rich medium in the bottom chambers serving as chemoattractant to promote invasion. In response to DOX-induced HuR silencing, invasion through Matrigel was significantly decreased in Mia.sh290 and Mia.sh700 cells (41% decrease and 56% decrease, respectively, *p* < 0.05). Invasion was increased with DOX-induced HuR overexpression in Mia.HuR cells (13.5-fold increase, *p* < 0.05), and unchanged in the control cell lines (Mia.CTRL and Mia.EV).

The results of both assays were reproduced in PL5 cells with transient knockdown or overexpression of HuR (Fig. S3). *In vitro* scratch assays showed a decrease in migration with HuR silencing, and an increase in migration with HuR overexpression (*p* < 0.01 for both). In Matrigel invasion assays, HuR silencing reduced invasion by 59% (*p* < 0.01), and HuR overexpression increased invasion 1.7-fold (*p* < 0.05). Taken together, these results demonstrate that both increases and decreases in HuR expression affect the invasive phenotype of PDA cells. This is in contrast to cell proliferation, where HuR overexpression had no effect.

### Lipidoid-delivered HuR siRNA suppresses established PDA xenograft growth

**Figure 6:**
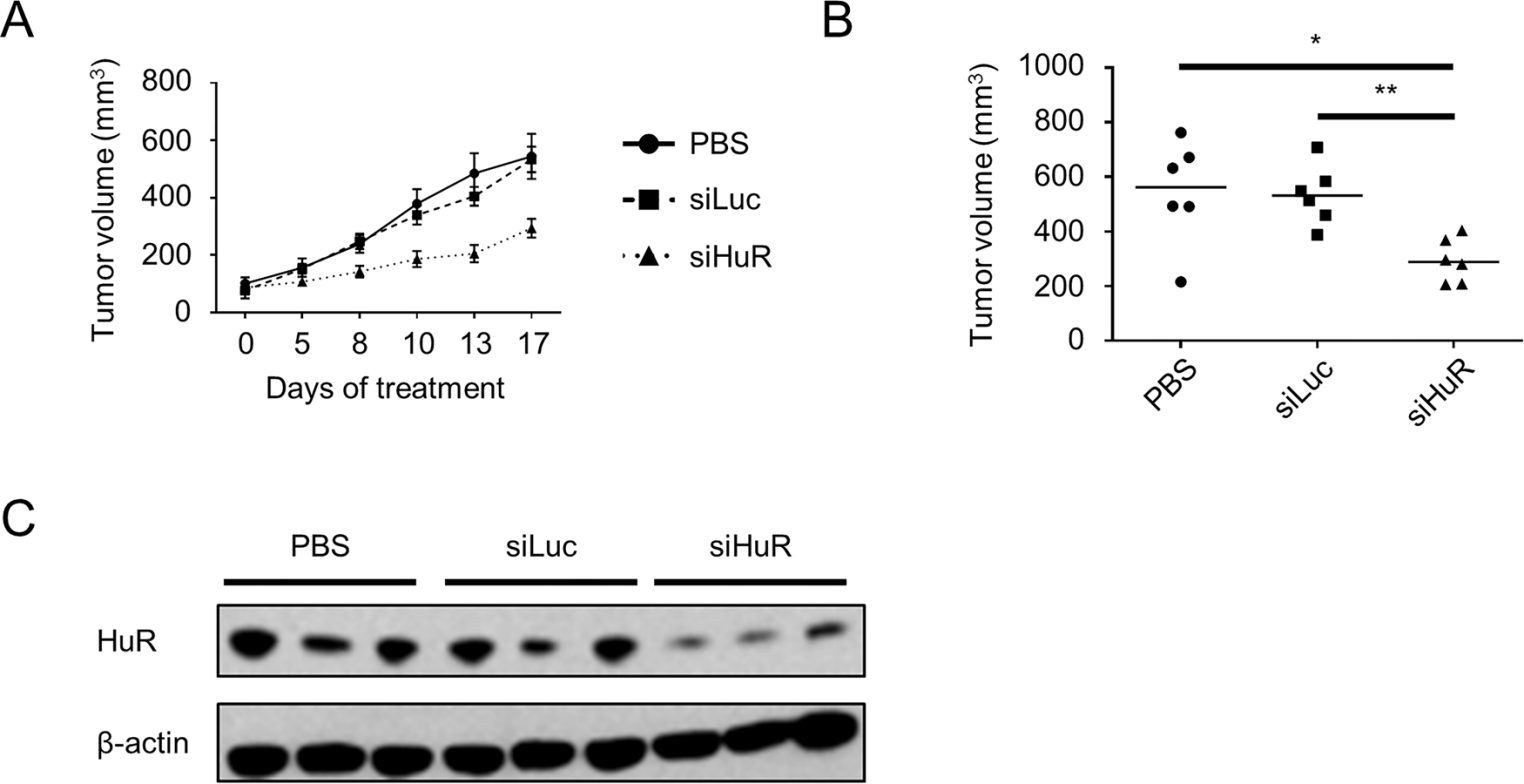
Lipidoid-delivered HuR siRNA suppresses established PDA xenograft growth **A.** Tumor volumes of MIA PaCa-2 xenografts that were allowed to grow to baseline size of ~100 mm^3^, then treated with PBS, siLuc, or siHuR for 17 days. Each data point represents the mean ± SEM (*n* = 6 per group). **B.** Plot of all tumor volumes on day 17 of treatment. Horizontal bars represent the median tumor volumes. **C.** Representative western blot of HuR protein expression in tumors harvested on day 17 of treatment. Beta-actin was used as normalization control. * = *p* < 0.05; ** = *p* < 0.01.

To test whether or not inhibition of HuR is a viable strategy to inhibit PDA growth *in vivo*, we performed a pilot experiment utilizing nude female mice subcutaneously injected in their hind flanks with MIA PaCa-2. At ~4 weeks post-injection, baseline tumor volumes were determined, and mice were randomly assigned to 3 groups for treatment with phosphate buffered saline (PBS) vehicle, firefly luciferase siRNA (siLuc), or HuR siRNA (siHuR). For intratumoral injection, siLuc and siHuR were encapsulated in the lipidoid nanoparticle 98N_12_-5, which has been previously shown to effectively deliver claudin-3 siRNA in an ovarian cancer xenograft model [58]. Mice were treated twice per week for 2 weeks, and tumor volumes were measured at the indicated time points (Fig. 6A). By day 8 of treatment, tumors in the siHuR treatment group were significantly smaller than tumors in either the PBS or siLuc treatment groups (*p* < 0.05). At the end of the pilot experiment (day 17), tumors in the siHuR group were 2-fold smaller than tumors in the PBS (*p* < 0.05) or siLuc (*p* < 0.01) groups (Fig. 6B). Western blotting of tumor lysates harvested at the end of the experiment validated HuR silencing in the siHuR treatment group (Fig. 6C).

### Induced HuR silencing suppresses PDA xenograft growth

**Figure 7:**
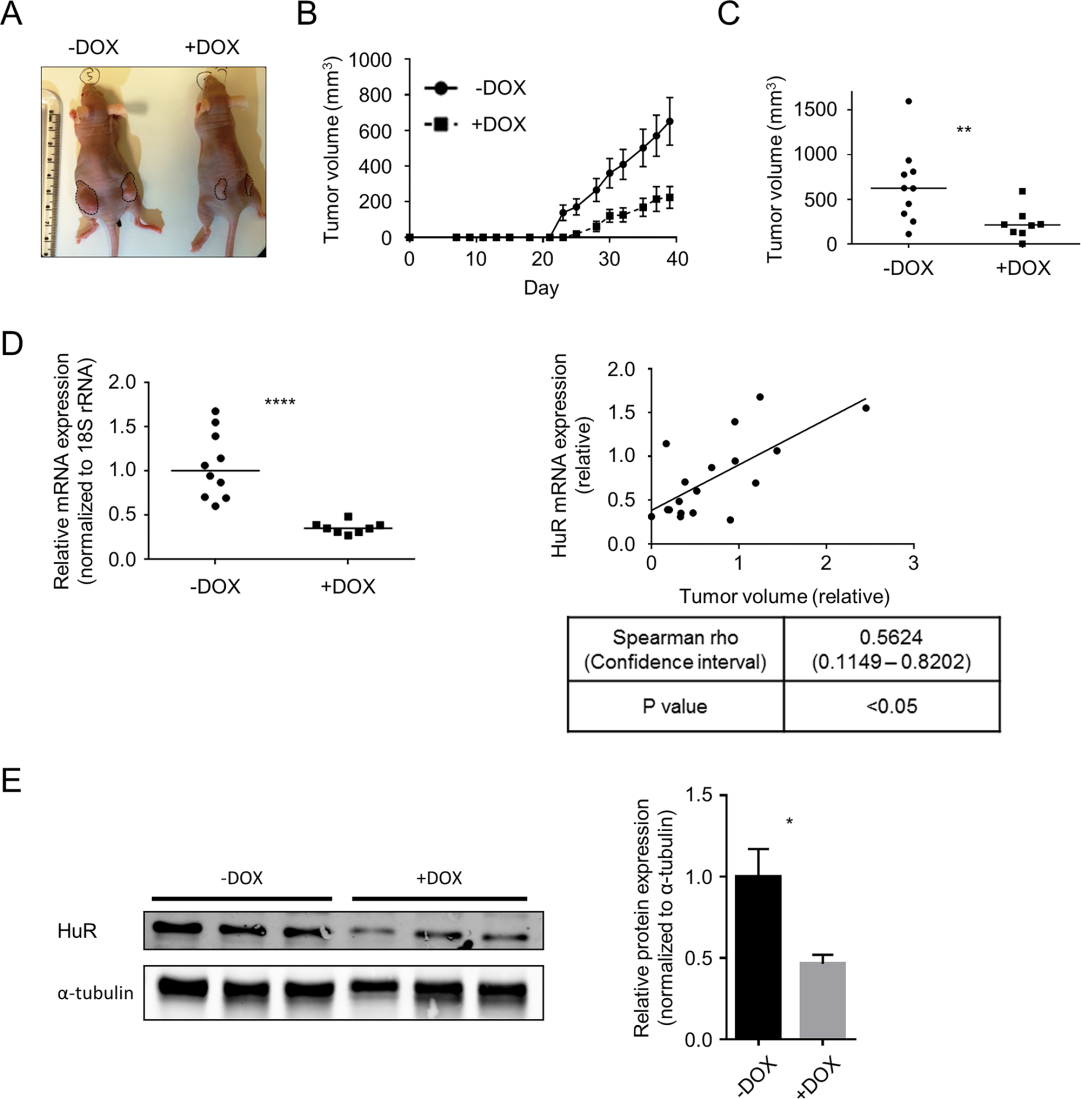
Induced HuR silencing suppresses PDA xenograft growth **A.** Representative images of subcutaneous Mia.sh290 tumors on the flanks of nude female mice, at the termination of the experiment (day 39). **B.** Tumor growth curves of Mia.sh290 xenografts. Mice were fed normal diet or 200 mg/kg DOX diet starting on day 0 (date of xenograft injection). Each data point represents the mean ± SEM (*n* = 10 for –DOX group, and *n* = 8 for +DOX group). **C.** Plot of all tumor volumes on the final day of the Mia.sh290 xenograft experiment (day 39). Horizontal bars represent the median tumor volumes. **D.** *Left*, qPCR analysis of HuR mRNA expression in all Mia.sh290 xenografts harvested on day 39. 18S rRNA was used as normalization control. Data were plotted relative to the mean HuR mRNA expression in the –DOX group. Horizontal bars represent the medians. *Right*, plot of HuR mRNA expression vs. tumor volume for all Mia.sh290 tumors. Data were plotted relative to the mean of the –DOX group. **E.** *Left*, representative western blot of HuR protein expression in Mia.sh290 tumors harvested on day 39. Alpha-tubulin was used as normalization control. *Right*, quantification of the western blot. Each bar represents the mean ± SEM (*n* = 3 per group). * = *p* < 0.05; ** = *p* < 0.01; **** = *p* < 0.0001.

Based on the result of our pilot experiment, we proceeded to test the effect of HuR inhibition on *in vivo* tumor growth utilizing one of our DOX-inducible HuR knockdown cell lines (Mia.sh290). We reasoned that this model would allow for more consistent and long-term inhibition of HuR in the tumors, compared to intratumoral injections of siRNA. Nude female mice were subcutaneously injected in their hind flanks with Mia.sh290 or Mia.CTRL cells. Half of the mice in each group were maintained on a DOX diet, and tumor volumes were measured three times per week. By the end of the experiment, the effect of DOX diet on the Mia.sh290 xenografts was profound, with 3-fold decrease in median tumor volume compared to xenografts in mice fed normal diet (623 mm^3^ vs. 212 mm^3^, *p* < 0.01) (Fig. 7A–7C). In contrast, DOX diet had no effect on tumor growth in the Mia.CTRL xenografts (Fig. S4).

In the Mia.sh290 xenografts, mRNA expression analysis by quantitative PCR (qPCR) demonstrated significant reduction of HuR mRNA in the DOX diet group (65% decrease compared to normal diet group, *p* < 0.0001) (Fig. 7D). Notably, we observed a significant correlation between tumor volume and HuR mRNA expression. That is, the largest tumors tended to have the highest HuR expression, whereas the smallest tumors tended to have the lowest HuR expression (Spearman rho = 0.5624, confidence interval 0.1149–0.8202, *p* < 0.05). Western blotting of tumor lysates validated HuR knockdown at the protein level (54% decrease in DOX diet group vs. normal diet group, *p* < 0.05) (Fig. 7E). Taken together, the two distinct xenograft models of HuR inhibition demonstrate that targeted inhibition of HuR may be a viable therapeutic strategy against PDA.

### HuR regulates multiple cancer-related pathways

**Figure 8:**
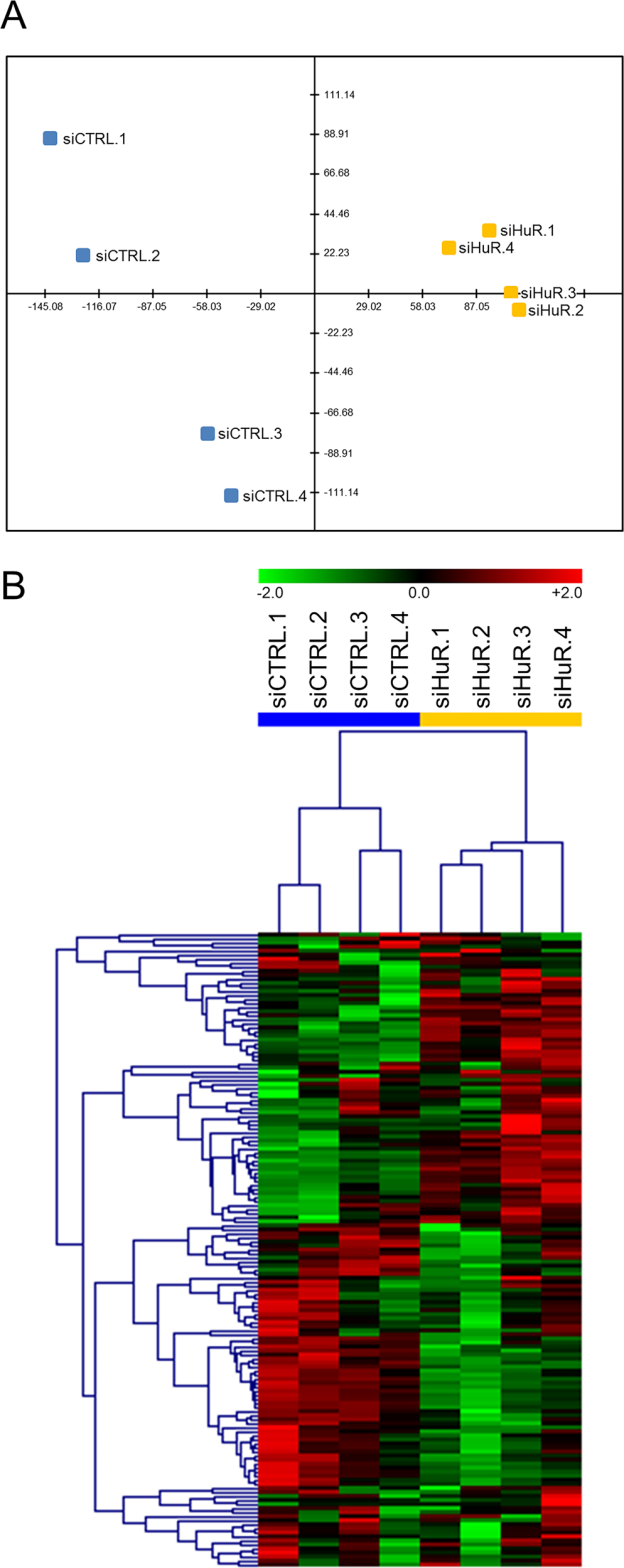
HuR regulates key cancer-related transcripts **A.** PCA of siCTRL and siHuR samples. X-axis = PC1 (variance explained: 47.8%), Y-axis = PC2 (variance explained: 16.3%). **B.** HCL analysis using Pearson correlation as the distance metric.

In order to better understand the mechanism by which HuR inhibition affects PDA phenotype in preclinical models, we performed gene expression analyses using the nCounter^®^ GX Human Cancer Reference Kit (NanoString Technologies, Seattle, WA), which allows for high-sensitivity and high-precision profiling of 230 genes (plus 6 housekeeping genes) known to be differentially expressed in various human cancers (see Table S1 for the full list of genes in the panel) [59]. Two separate experiments were performed. In the first experiment, MIA PaCa-2 cells were transiently transfected with control siRNA (siCTRL) or HuR siRNA (siHuR) for 72 hours, and total RNA samples were isolated and analyzed (4 biological replicates each). Multivariate statistical analysis of the filtered and normalized dataset (containing 157 genes) revealed that the replicates for the siHuR group were clearly separated from the replicates for the siCTRL group (Figs. 8 and S5 and Table 1). In more detail, the principal component analysis (PCA) graph is a two-dimensional representation with each sample being projected on each axis based on its transcript profile (Fig. 8A). The fact that the siHuR samples are separated from the siCTRL samples indicates that the analyzed transcript profile changes significantly after HuR knockdown. In the case of hierarchical clustering (HCL) analysis, the dendrogram shows how well samples, or clusters of samples, are correlated with each other (Fig. 8B). As shown, the siHuR and siCTRL groups arranged in two distinct clusters, supporting the results of our PCA analysis. Finally, significance analysis of microarrays (SAM) was used to identify transcripts with significantly different expression after HuR knockdown, and found 87 such transcripts (out of 157, or 55%) (Fig. S5 and Table 1). In the siHuR samples, 43 transcripts (27%) were significantly upregulated, and 44 transcripts (28%) were significantly downregulated, compared to siCTRL. The 5 upregulated transcripts with the greatest fold change were *TGFB1* (2.46-fold), *L1CAM* (2.31-fold), *MMP1* (1.83-fold), *FGFR3* (1.77-fold), and *PTK7* (1.75-fold). The 5 downregulated transcripts with the greatest fold change were *CDK4* (0.56-fold), *EPS8* (0.59-fold), *NRAS* (0.61-fold), *BRCA2* (0.62-fold), and *CHEK1* (0.63-fold). These transcripts encompassed those that are regulated by HuR directly (i.e. HuR binds to the transcripts and directly regulates their stability and/or translation), as well as indirectly (i.e. HuR does not bind to the transcripts, but their expression is altered as a downstream effect of HuR’s direct regulation of other targets).

**Table 1:**
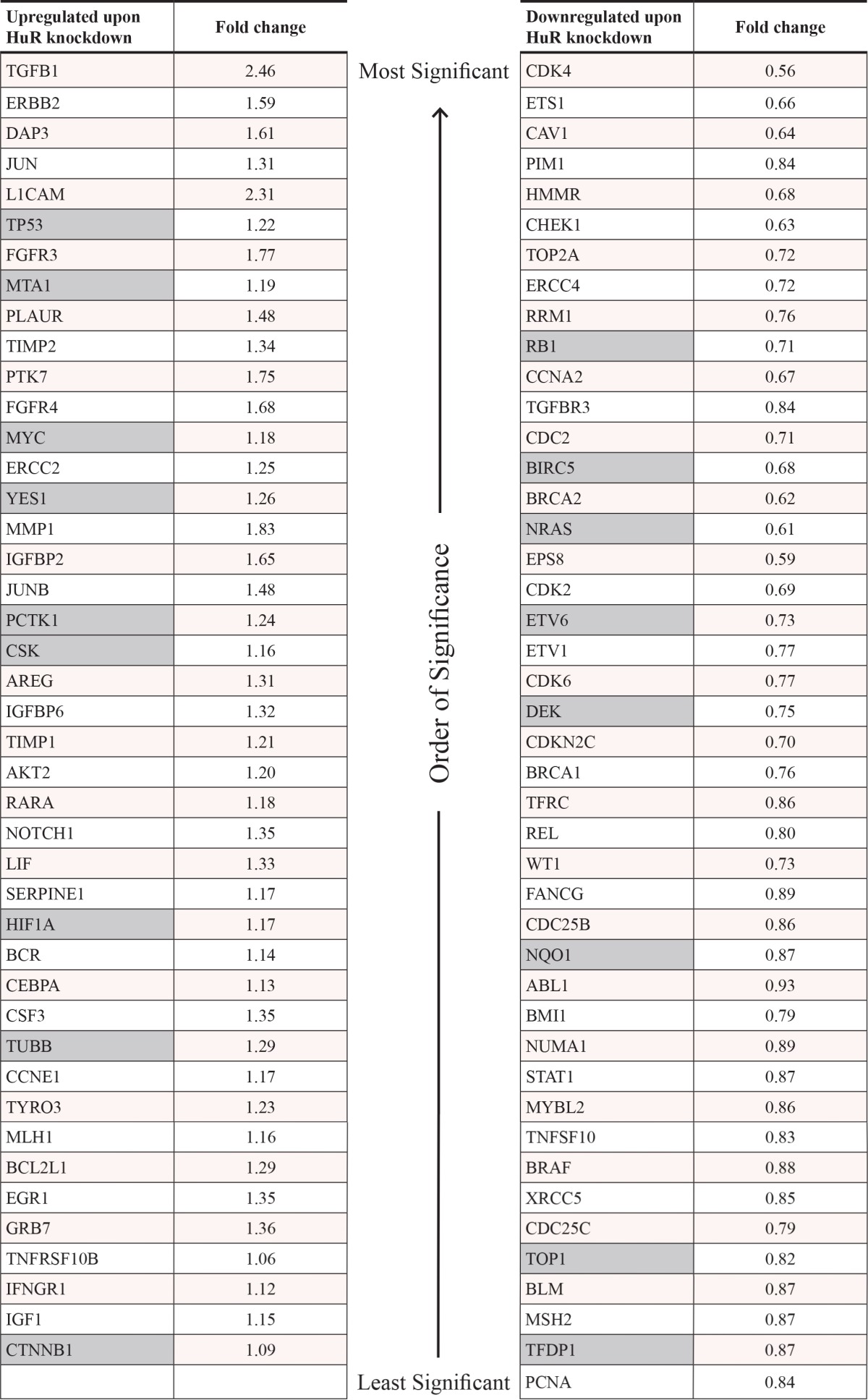
HuR-regulated transcripts identified by NanoString nCounter^®^ analyses

Full list of transcripts that were upregulated (*left*) or downregulated (*right*) in MIA PaCa-2 cells transfected for 72 hours with HuR siRNA, compared to cells transfected with control siRNA, with the associated fold changes. All fold changes are significant (*p* < 0.05), with the list of transcripts in each column sorted in the order of significance (most significant at the top; least significant at the bottom). Gray cells indicate transcripts that were identified as direct HuR targets (i.e. transcripts that were significantly enriched in HuR mRNP-IP samples compared to control IgG mRNP-IP samples, as described in Table S2). False discovery rate (FDR) = 0% for all transcripts.

In order to distinguish the direct and indirect targets of HuR, we performed messenger ribonucleoprotein-immunoprecipitation (mRNP-IP) in MIA PaCa-2 cells to isolate mRNA transcripts that were directly bound to HuR (2 biological replicates). For comparison, mRNP-IP with isotype control immunoglobulin G (IgG) was performed (2 biological replicates). Fig. S6 shows the western blot and qPCR performed as quality control steps to validate that the mRNP-IP was specific and successful. The input samples (cytoplasmic lysates) were all positive for HuR protein and alpha-tubulin protein, the HuR IP samples were positive for HuR protein but not alpha-tubulin protein, and the control IgG IP samples were negative for both HuR protein and alpha-tubulin protein. qPCR showed that compared to RNAs isolated from control IgG mRNP-IP samples, RNAs isolated from HuR mRNP-IP samples were significantly enriched in the transcript of deoxycytidine kinase (*DCK*), which has been previously demonstrated by our group to be a direct binding target of HuR protein [30]. When gene expression analysis for these mRNP-IP RNA samples was performed using the nCounter^®^ GX Human Cancer Reference Kit, we were able to identify 35 transcripts (out of 236, or 15%) that were enriched in the HuR mRNP-IP RNA samples compared to the IgG mRNP-IP RNA samples (i.e. direct targets of HuR) (Table S2). The 5 most enriched transcripts were *SFPQ* (1,289-fold), *BIRC5* (1,273-fold), *TP53* (1,069-fold), *CTNNB1* (788-fold), and *CD44* (758-fold). By cross-referencing these 35 transcripts with the 87 aforementioned transcripts, we were able to identify a total of 17 transcripts that were both direct targets of HuR, and differentially expressed upon HuR silencing (9 upregulated and 8 downregulated transcripts in siHuR vs. siCTRL) (Table 1). Notably, 31 out of the 35 direct targets of HuR were previously independently validated, wherein various high-throughput targeting technologies (e.g. photoactivatable-ribonucleoside-enhanced crosslinking and immunoprecipitation or PAR-CLIP) identified the HuR “target-ome” in a modified human embryonic kidney (HEK) cell line [60].

Functional annotation enrichment analysis using the Database for Annotation, Visualization, and Integrated Discovery (DAVID) revealed the biological processes that were significantly enriched based on the list of HuR-regulated transcripts (Table S3). Among the 43 genes upregulated with HuR knockdown, the top enriched biological processes were related to transcriptional regulation, nucleotide synthesis, and nucleotide metabolism. Among the 44 genes downregulated with HuR knockdown, the top enriched biological processes were related to cell cycle, cell proliferation, DNA repair, and apoptosis. Among the 35 genes whose transcripts are directly bound by HuR, the top enriched biological processes were related to cell cycle, cell proliferation, and apoptosis. Taken together, these results suggest that therapeutic targeting of HuR can affect multiple essential biological processes at once, by disrupting HuR’s regulation of numerous cancer-related transcripts.

## DISCUSSION

Despite the discovery of multiple genomic alterations that give rise to PDA, attempts to exploit these lesions (e.g. oncogenic *RAS* activation) for either early detection or treatment have so far been unsuccessful in the clinical setting [2–5, 7, 61]. In order to change the paradigm of PDA management for the better, novel strategies that venture outside of the traditional “gene-centric” approach may be necessary. This perspective prompted us to investigate a relatively understudied, but highly relevant, aspect of cancer biology – post-transcriptional regulation by RBPs. While others have studied the RBP HuR in the context of other cancer types, we are the first to study this regulatory protein in preclinical models of PDA. The findings from this study complement previous studies by our group that cast HuR as an intriguing therapeutic target in PDA [25, 53, 54, 62].

In the present study, we demonstrated that silencing HuR expression significantly inhibits PDA proliferation *in vitro* (Figs. 2, 3, and S2) and *in vivo* (Figs. 6 and 7). However, in contrast to the majority of published results, HuR *overexpression* did not enhance PDA proliferation in our cell culture models (Figs. 2, 3, and S2). We speculate that in PDA, HuR’s contribution to proliferative potential is maximal (i.e. saturated) at baseline expression levels. In fact, too much expression/activity of HuR has been shown to have detrimental effects on cancer cells. For example, Gubin et al. demonstrated that exogenous HuR overexpression in the MDA-MB-231 triple-negative breast cancer cell line enhanced cell proliferation *in vitro*, but dramatically impaired tumor growth *in vivo* in an orthotopic xenograft model [57]. Moreover, although HuR is normally a promoter of cell survival, exposure of cells to lethal stress (in which cell death is unavoidable) has been shown to irreversibly convert HuR into a promoter of apoptosis [63]. These observations support the notion that HuR’s effect on the cancer phenotype is most likely both tissue- and context-dependent. In comparison to HuR’s effect on cell proliferation, both HuR overexpression and silencing affected PDA invasiveness in *in vitro* experiments (Figs. 4, 5, and S3).

Even though our findings support a strong role for HuR in PDA tumorigenesis, the presented data should be interpreted in the context of the preclinical models used in this study. For instance, the proof-of-principle studies described herein were performed in a limited number of immortalized, established PDA cell lines. Commercial cell lines perform robustly under many experimental conditions and exhibit hallmark molecular aspects of PDA (e.g. *KRAS* and *TP53* mutations), thereby functioning as convenient model systems; yet, their generalizability to primary and metastatic PDAs in patients is difficult to determine [64, 65]. To that end, we are in the process of establishing conditionally-reprogrammed cells and organoids derived from PDA patient samples, which should serve as complementary alternatives to established cell lines [66, 67].

Additionally, subcutaneous heterotopic xenografts have limitations as an *in vivo* model for PDA [68]. Tumor metastases cannot be properly studied with subcutaneous xenografts, and the hallmark desmoplastic reaction observed in most human PDAs do not develop in this preclinical model [2, 4, 68–70]. Thus, we are currently in the long-term process of utilizing an established genetically engineered mouse model (GEMM) of PDA – *Kras^LSL-G12D/+^*; Trp53*^LSL-R172H/+^*; Cre** (KPC) – as a background to manipulate pancreas-specific HuR expression (Sawicki et al., unpublished) [71]. In the present study, HuR overexpression did not affect the growth of established PDA *in vitro*, in the context of an HuR network that is already highly engaged in PDA cell lines (Figs. 2, 3, and S2). However, we hypothesize that HuR overexpression may cooperate with *KRAS* and *TP53* mutations to promote pathogenesis from normal cells (where HuR activity is typically minimal at baseline) to pre-cancerous pancreatic intraepithelial neoplasia (PanIN) and eventually PDA. Conversely, we hypothesize that crossing a pancreas-specific HuR deletion GEMM with KPC GEMM will dramatically retard or even eliminate pancreatic tumorigenesis. With the proper controls, these GEMMs will provide an ideal setting to study HuR within the context of an intact immune system and a more relevant tumor microenvironment. In particular, these GEMMs will enable studies into HuR’s role in every stage of tumor development – from the early stages of tumor initiation to the late stages of metastatic invasion.

Consistent with previous mechanistic studies, we have demonstrated here that HuR directly and indirectly regulates numerous cancer-related transcripts (Fig. 8 and Table 1). These transcripts play critical roles in multiple essential biological processes related to cell cycle, DNA repair, apoptosis, and nucleotide metabolism, among others, and emphasize the notion that therapeutic inhibition of HuR alone can simultaneously affect all of these pathways. In fact, previous studies revealed that HuR regulates clusters of genes, referred to as *regulons*, in order to efficiently affect whole pathways [72]. In light of HuR’s broad influence over diverse critical pro-survival pathways, HuR inhibition provides a unique therapeutic opportunity observed with just a handful of master regulators of cancer biology (e.g. *MYC*) [73]. In conventional targeted therapeutic strategies, only one gene or one pathway is typically affected at a time. Cancer cells rely on intrinsic redundancy in cellular signaling, and invoke any number of chemoresistance mechanisms to overcome therapies that fit under the umbrella of “precision medicine” [74]. In contrast, HuR inhibition offers a new *global synthetic lethal* approach, in which multiple core signaling pathways are targeted at once, leaving the cancer cells with no viable compensatory mechanisms.

To date, the best-characterized small molecule inhibitor of HuR is the chrysanthone-like compound MS-444 [75]. Despite its success as a lead compound in preclinical models, issues with its stability and bioavailability *in vivo* may hinder its clinical utility (Blanco et al., unpublished). As such, we have begun to explore several alternative strategies for HuR inhibition. In the present study, we were able to demonstrate effective HuR knockdown and reduction of tumor growth utilizing HuR siRNA intratumorally delivered by lipidoid nanoparticles (Fig. 6). However, a clinically relevant therapy for PDA must have systemic activity in order to treat microscopic and macroscopic metastases, which typically drive the clinical course of the disease in patients [76]. Studies that involve systemic therapeutic strategies to inhibit HuR will need to address toxicity and define a therapeutic window. It has been previously reported that postnatal global deletion of HuR in mice has no apparent effect on quiescent stem cells and differentiated cells [77]. However, rapidly proliferating progenitor cells in the thymus, bone marrow, and intestine underwent apoptosis, and the mice eventually died due to atrophy of hematopoietic organs and obstructive enterocolitis. This suggests that any HuR inhibitor that acts systemically may very well, at minimum, be accompanied by toxicities similar to those seen with conventional chemotherapeutics (e.g. nausea/vomiting/diarrhea, immunosuppression). In order to minimize potential systemic toxicities, we are currently developing a systemic siRNA-based strategy that utilizes a DNA scaffold, which may be functionalized with targeting moieties that preferentially direct the siRNA to PDA cells.

Although a therapeutic window of opportunity may be realized with generalized HuR inhibition due to the fact that its regulatory targets are commonly active in cancer-associated survival pathways (as compared to normal cells), we believe that inhibiting HuR’s most critical binding interactions may very well improve specificity without compromising therapeutic efficacy. This may be theoretically accomplished by precisely disrupting the interaction between HuR and the specific AREs present on selected target transcripts. This approach should mitigate the disruption of HuR’s regulation of house-keeping transcripts (which contain different AREs) that are essential for normal biological processes. Specifically, we have begun to develop decoy RNA oligonucleotides that contain sequence motifs complementary to the 3′ UTRs of cancer-specific HuR binding targets (e.g. *WEE1*) [54]. These “suicide UTRs,” or “sUTRs,” can theoretically compete with HuR for these regulatory binding sites (essentially acting as RNA sponges), and thereby antagonize HuR-directed pro-survival pathways.

To summarize, we provide proof-of-principle evidence that targeted HuR inhibition impairs the malignant phenotype of PDA cells in both *in vitro* and *in vivo* preclinical models. Subsequent translational studies, utilizing more clinically relevant model systems, will further determine the utility of targeting HuR to treat PDA as well as other cancers.

## MATERIALS AND METHODS

### Cell culture

MIA PaCa-2 human pancreatic ductal adenocarcinoma (PDA) cell line was purchased from American Type Culture Collection (ATCC, cat. #CRL-1420). PL5 human PDA cell line was kindly provided by S. E. Kern (Johns Hopkins University, Baltimore, MD). Both cell lines were cultured in Dulbecco’s modified Eagle medium (DMEM; Life Technologies, cat. #11965-084) supplemented with 10% Tet System Approved Fetal Bovine Serum (FBS; Clontech Laboratories, cat. #631106) and 2 mM L-glutamine (Gemini Bio-Products, cat. #400-106). Cells were routinely passaged and cultured at 37°C in a humidified incubator with 5% CO_2_.

### Generation of doxycycline (DOX)-inducible MIA PaCa-2 cell lines

#### DOX-inducible HuR knockdown cell lines

To generate short hairpin RNA (shRNA)-expressing plasmids, shRNA-encoding oligonucleotides targeting HuR (referred to as sh290 and sh700) were cloned into the Tet-pLKO-puro lentiviral plasmid (Addgene, cat. #21915) as previously described [78]. The targeted sequences were as follows: sh290 = sense 5′-GCAGCAUUGGUGAAGUUGAAUCU-3′, antisense 5′-AGAUUCAACUUCACCAAUGCUGC-3′; sh700 = sense 5′-GCCCAUCACAGUGAAGUUUGCA-3′, antisense 5′-UGCAAACUUCACUGUGAUGGGC-3′. Lentiviruses were generated and transduced into MIA PaCa-2 at multiplicity of infection (MOI) = 1, and selection was performed with 1 μg/ml puromycin dihydrochloride (Life Technologies, cat. #A11138-03) to isolate clones of Mia.CTRL (transduced with empty vector lentivirus), Mia.sh290, and Mia.sh700.

#### DOX-inducible HuR overexpression cell lines

Tet-On 3G Inducible Expression System (Clontech Laboratories, cat. #631165) was used according to manufacturer’s instructions to generate MIA PaCa-2 cells stably transfected with pCMV-Tet3G plasmid (Mia.pCMV-Tet3G). HuR complementary DNA (cDNA) was cloned into pTRE3G-mCherry plasmid using In-Fusion HD Cloning Plus (Clontech Laboratories, cat. #638909). Mia.pCMV-Tet3G cells were then stably transfected with either empty vector (pTRE3G-mCherry) or HuR overexpression vector (pTRE3G-mCherry-HuR) to generate Mia.EV and Mia.HuR cell lines, respectively.

For DOX induction, cells were treated with 2 μg/ml DOX hyclate (Sigma-Aldrich, cat. #D9891) for the indicated times.

#### DNA and small interfering RNA (siRNA) transfections

For transient HuR overexpression studies, the coding region of the human *ELAVL1* gene was subcloned from pCMV6-XL5 *ELAVL1* plasmid (OriGene, cat. #SC119271) into pcDNA3.1/Zeo^(+)^ plasmid (Life Technologies, cat. #V860–20), and transfected into PDA cells. Transfection with empty vector served as control. Transient HuR knockdown studies were performed by transfecting cells with a custom-made HuR siRNA oligonucleotide (GE Dharmacon; sense 5′-CCAUUAAGGUGUCGUAUGCUCUU-3′, antisense 5′-UUGAGCAUACGACACCUUAAUGG-3′). Transfection with ON-TARGET *plus* Non-targeting Control siRNA (GE Dharmacon, cat. #D-001810-01-05) was used as negative control. All transfections were performed using Lipofectamine 2000 (Life Technologies, cat. #11668) according to manufacturer’s instructions.

#### Messenger RNA (mRNA) expression analysis

Total RNA was extracted using TRIzol Reagent (Life Technologies, cat. #15596) according to manufacturer’s instructions. cDNA was synthesized from 1 μg of total RNA using random primers and MultiScribe Reverse Transcriptase (Life Technologies, cat. #4368813). Quantitative PCR (qPCR) analysis was performed using Applied Biosystems 7500 Fast Real-Time PCR System (Life Technologies, cat. #4351107) and SYBR Select Master Mix (Life Technologies, cat. #4472908). HuR mRNA expression was detected with the following primer set: sense 5′-GCTCGGTCTACTCAGGCATC-3′, antisense 5′-CCAGTCCAGGAGCCTAATGA-3′. 18S rRNA expression was used for normalization, and was detected with the following primer set: sense 5′-GTAACCCGTTGAACCCCATT-3′, antisense 5′-CCATCCAATCGGTAGTAGCG-3′. Relative quantification was performed using the 2^−ΔΔCt^ method, as previously described [79].

#### Western blot analysis

For total protein extraction, cell pellets were resuspended in radioimmunoprecipitation assay (RIPA) buffer supplemented with 1X protease inhibitor cocktail, 1 mM sodium orthovanadate, and 2 mM phenylmethanesulfonylfluoride (PMSF) (Santa Cruz Biotechnology, cat. #sc-24948). Samples were placed on ice for 5 minutes, then centrifuged at 16,000 × *g*, at 4°C, for 30 minutes. The supernatants were recovered, and protein concentrations were determined using Pierce BCA Protein Assay Kit (Life Technologies, cat. #23225). The protein samples were mixed with Laemmli sample buffer, heated at 95°C for 10 minutes, then size-fractionated on homemade 10% polyacrylamide gels. The samples were then transferred to Immun-Blot low fluorescence PVDF membranes (Bio-Rad Laboratories, cat. #162–0264) for 2 hours at 4°C. The membranes were blocked for 1 hour at room temperature using Odyssey Blocking Buffer (LI-COR Biosciences, cat. #927–40000), then probed overnight at 4°C with mouse monoclonal antibodies against HuR (Santa Cruz Biotechnology, cat. #sc-5261), alpha-tubulin (Life Technologies, cat. #32-2500), and/or beta-actin (Sigma-Aldrich, cat. #A5441) at dilutions of 1:2,000, 1:10,000, and 1:5,000, respectively, in Odyssey Blocking Buffer supplemented with 0.1% Tween 20 (Fisher Scientific, cat. #BP337). Following washes with TBST, the membranes were incubated for 1 hour at room temperature with IRDye 800CW Goat anti-Mouse Immunoglobulin G (IgG) (LI-COR Biosciences, cat. #926–32210) using dilution of 1:20,000 in Odyssey Blocking Buffer supplemented with 0.1% Tween 20 and 0.01% sodium dodecyl sulfate (SDS; Affymetrix, cat. #77504). Following washes with TBST, the membranes were scanned using Odyssey Infrared Imaging System (LI-COR Biosciences, model #9120) for target protein detection. Signal quantification was performed using Odyssey Infrared Imaging System software.

#### Cell proliferation assay

Cells were seeded in 96-well culture plates at 250 cells/well, and allowed to grow for 0–10 days in media containing 0 or 2 μg/ml DOX. At each indicated time point, cells were rinsed twice with Dulbecco’s phosphate buffered saline (DPBS; Life Technologies, cat. #14190), and lysed with deionized water. Double-stranded DNA (dsDNA) was stained by Quant-iT PicoGreen dsDNA reagent (Life Technologies, cat. #P7581), and fluorescence intensity was measured by a microplate reader (Tecan, part #F129015) using excitation wavelength of 485 nm and emission wavelength of 535 nm. Data were plotted relative to day 0 to provide estimates of cell proliferation based on dsDNA content.

#### Soft agar colony formation assay

The experiment was performed as previously described, with slight modifications [80]. Briefly, the base agarose layer was prepared in 6-well culture plates by pouring 2 ml of base agarose mixture comprised of 1X DMEM, 10% FBS, and 0.5% agarose (Affymetrix, cat. #32802). Cells were prepared in top agarose mixture comprised of 1X DMEM, 10% FBS, and 0.35% agarose, and poured over the solidified base agarose layer at final seeding density of 5,000 cells/well. After solidification of the top layer, 2 ml of growth media with 0 or 2 μg/ml DOX were added to each well, and samples were placed in a 37°C incubator. Cells were allowed to incubate for 4 weeks, with the overlaid media and DOX exchanged thrice weekly. At the termination of the experiment, samples were rinsed twice with DPBS and fixed with 3.7% formaldehyde (Sigma-Aldrich, cat. #252549) for 10 minutes. After two more washes with DPBS, cells were stained with 0.01% crystal violet (Santa Cruz Biotechnology, cat. #sc-207460) for 1 hour. Photographs were taken using EVOS FL Imaging System, and colonies were counted using ImageJ (National Institutes of Health, Bethesda, MD). To exclude background, only colonies > 10,000 μm^2^ were counted.

#### *In vitro* scratch assay

Cells were pre-treated with 0 or 2 μg/ml DOX for 5 days, then seeded in 6-well culture plates and allowed to grow to confluence. The cell monolayer was scratched in a straight line with a p200 pipet tip, then rinsed twice with DPBS to remove debris. Fresh culture medium was added, with 2 μg/ml DOX in the appropriate wells. Every 12 hours, photographs of the scratches were taken using EVOS FL Imaging System. The mean width of each scratch was measured using ImageJ. Data were plotted relative to the 0 hour time point.

#### Matrigel invasion assay

The experiment was performed as described in Corning’s Cell Invasion Assay protocol (available online at the following URL: http://csmedia2.corning.com/LifeSciences/media/pdf/protocol_DL_031_Cell_Invasion_Assay.pdf). Briefly, permeable supports (Corning, cat. #353097) were coated with 200 μg/ml Matrigel basement membrane matrix (Corning, cat. #354234), and inserted in 24-well companion plates (Corning, cat. #353504). Cell suspensions were prepared in serum-free DMEM, and seeded in invasion chambers at 25,000 cells/chamber. DMEM with 20% FBS was added to each well as chemoattractant. Samples were incubated at 37°C for 24 hours to allow for cell migration through the Matrigel. Non-invading cells on the apical surface of the Matrigel-coated supports were removed with cotton swabs, and cells that had migrated to the lower surface of the supports were stained using Differential Quik Stain Kit (Polysciences, cat. #24606). Photographs were taken, and cells were counted using ImageJ. Samples were prepared in triplicate, and 5 fields were photographed per sample (at 20X magnification).

### Mouse xenografts

Mouse protocols were approved by the Thomas Jefferson University Institutional Animal Care and Use Committee.

#### siHuR lipidoid nanoparticle study

We encapsulated siRNAs in the lipidoid nanoparticle 98N_12_-5, as previously described [58]. The targeted sequences were as follows: firefly luciferase siRNA (siLuc) = sense 5′-CUUACGCUGAGUACUUCGA-3′, antisense 5′-UCGAAGUACUCAGCGUAAG-3′; HuR siRNA (siHuR) = sense 5′-GCGUUUAUCCGGUUUGACA-3′, antisense 5′-UGUCAAACCGGAUAAACGC-3′. Nine 6-week-old, female, athymic nude mice (Harlan Laboratories, cat. #6904F) received subcutaneous injections of 2 × 10^6^ MIA PaCa-2 cells in both flanks. Cells were prepared in 100 μl solution comprised of 80% DPBS and 20% Matrigel (Corning, cat. #356237). Tumors were allowed to grow to mean tumor volume of ~100 mm^3^, whereupon mice were randomly assigned to 3 groups. Mice received intratumoral injections of 1) PBS; 2) siLuc lipidoid nanoparticle; or 3) siHuR lipidoid nanoparticle, following the treatment regimen of 25 mg/kg, twice per week for 2 weeks. Tumors were measured at the indicated time points using an electronic caliper, and tumor volumes were calculated using the formula Volume = Length × Width^2^/2. Upon termination of the experiment, mice were euthanized using carbon dioxide inhalation followed by cervical dislocation, and tumors were harvested.

#### DOX-inducible HuR knockdown study

6-week-old, female, athymic nude mice received subcutaneous injections of 2 × 10^6^ Mia.sh290 cells or 1.5 × 10^6^ Mia.CTRL cells in both flanks (10 mice per cell line). Cells were prepared in 100 μl solution comprised of 80% DPBS and 20% Matrigel. Starting on the day of injection, 5 mice were fed 200 mg/kg DOX diet (Bio-Serv, cat. #S3888), while the other 5 mice were maintained on control diet (Bio-Serv, cat. #S4207). Tumors were measured three times per week using an electronic caliper, and tumor volumes were calculated using the formula Volume = Length × Width^2^/2. For the Mia.sh290 xenografts, the experiment was terminated on day 39, when one of the tumors surpassed 1,500 mm^3^ (a pre-defined requirement for termination). For the Mia.CTRL xenografts, the experiment was terminated on day 72, upon clear evidence that DOX diet had no significant effect on tumor growth over a prolonged time period. Upon termination of the experiment, mice were euthanized using carbon dioxide inhalation followed by cervical dislocation, and tumors were harvested.

#### Messenger ribonucleoprotein-immunoprecipitation (mRNP-IP)

mRNP-IPs were performed as previously described [81]. Briefly, MIA PaCa-2 cytoplasmic lysates were obtained using CelLytic NuCLEAR Extraction Kit (Sigma-Aldrich, cat. #NXTRACT) according to manufacturer’s instructions, with the modification of supplementing with 100 U/ml RNase inhibitor (Life Technologies, cat. #N8080119) to preserve RNA integrity. HuR protein and its bound mRNA cargo were immunoprecipitated by incubating the cytoplasmic lysates with mRNP-IP-grade HuR antibody (MBL International Corporation, cat. #RN004P) or isotype control IgG (Santa Cruz Biotechnology, cat. #sc-2027) pre-coated to Protein A-Sepharose beads (Sigma-Aldrich, cat. #P9424). HuR was digested with proteinase K (Life Technologies, cat. #AM2546), and the released mRNA transcripts were purified with TRIzol Reagent for further analysis.

#### NanoString nCounter^®^ assay, data normalization and filtering, multivariate statistical analysis, and pathway analysis

For the identification of cancer-related transcripts that were differentially expressed between MIA PaCa-2 cells transfected with control siRNA or HuR siRNA, 200 ng each of total RNA samples were analyzed with the nCounter^®^ GX Human Cancer Reference Kit (NanoString Technologies; see Table S1 for the complete gene list) according to manufacturer’s protocols [59]. Six positive hybridization controls that do not correspond to any known transcript sequence were added at fixed amounts into each sample. Eight probes served as negative controls that were used to estimate the background noise (average plus three standard deviations for each sample). Transcripts with expression lower than this threshold in four or more samples were filtered out of the analysis. The remaining 157 transcripts were then normalized based on the geometric mean of the positive controls for each sample. From the resulting table, the three transcripts with the lowest coefficient of variation (CoV) were chosen (CoV < 7%): *FOSL2*, *TNFRSF10B*, and *ABL1*. These transcripts were flagged as “housekeeping genes,” and the values in each sample were further normalized based on the consideration that the geometric mean of the expressions of these transcripts should remain unchanged across all samples. Transcript expression was also standardized before multivariate statistical analysis.

Principal component analysis (PCA), hierarchical clustering (HCL; Pearson correlation metric), and significance analysis of microarrays (SAM) were performed using the TM4-MeV statistical analysis software, as previously described [82–84]. Pathway enrichment was run using the Database for Annotation, Visualization, and Integrated Discovery (DAVID) [85, 86]. Specifically, enrichment for biological process (BP) terms of gene ontology (GO) was sought. The background list for the enrichment analysis was the full list of 236 genes included in the nCounter^®^ GX Human Cancer Reference Kit.

For the identification of the cancer-related transcripts that HuR directly binds, 200 ng each of HuR and control IgG mRNP-IP RNA samples were analyzed with the nCounter^®^ GX Human Cancer Reference Kit, as above. Data filtering and positive control normalization were also performed as described above. To identify binders, the average of the normalized counts of the two HuR mRNP-IP replicates had to be at least twice the maximum of the normalized counts of the two control IgG mRNP-IP replicates. Fold enrichment for each binder was calculated as the ratio of its average expression in the HuR mRNP-IP samples relative to the average signal of the non-binders.

Further details in regards to statistics are available upon request.

## SUPPLEMENTARY FIGURES AND TABLE






