# Supplementary material for: Targeting the mRNA-binding protein HuR impairs malignant characteristics of pancreatic ductal adenocarcinoma cells
Source: Oncotarget. 2015 Jul 25;6(29):27312–31. doi: 10.18632/oncotarget.4743 (PMC4694992; doi:10.18632/oncotarget.4743)
Supplement: Supplementary file 1 [file oncotarget-06-27312-s001.pdf]

SUPPLEMENTARY FIGURES AND TABLES

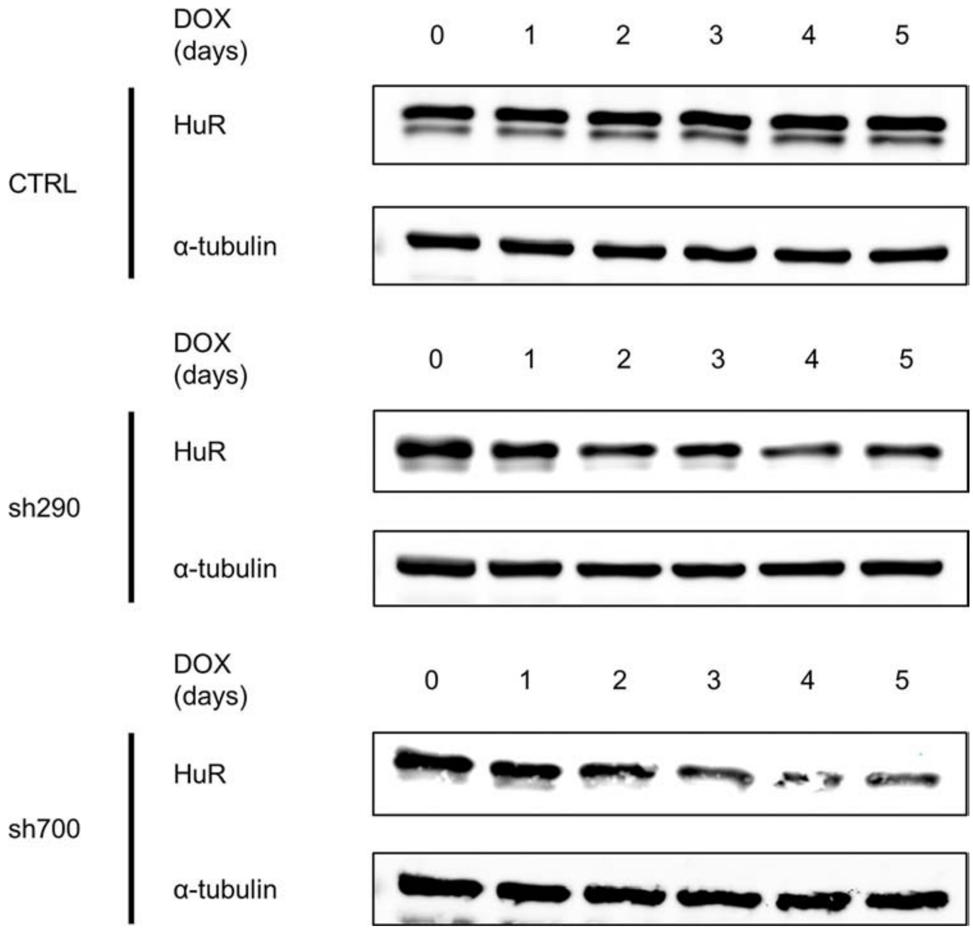

Supplementary Figure S1: Time course of DOX-induced HuR silencing. Western blot of HuR protein expression in Mia.CTRL, Mia.sh290, and Mia.sh700 cells treated with 2 µg/ml DOX for the indicated time points. Alpha-tubulin was used as normalization control.

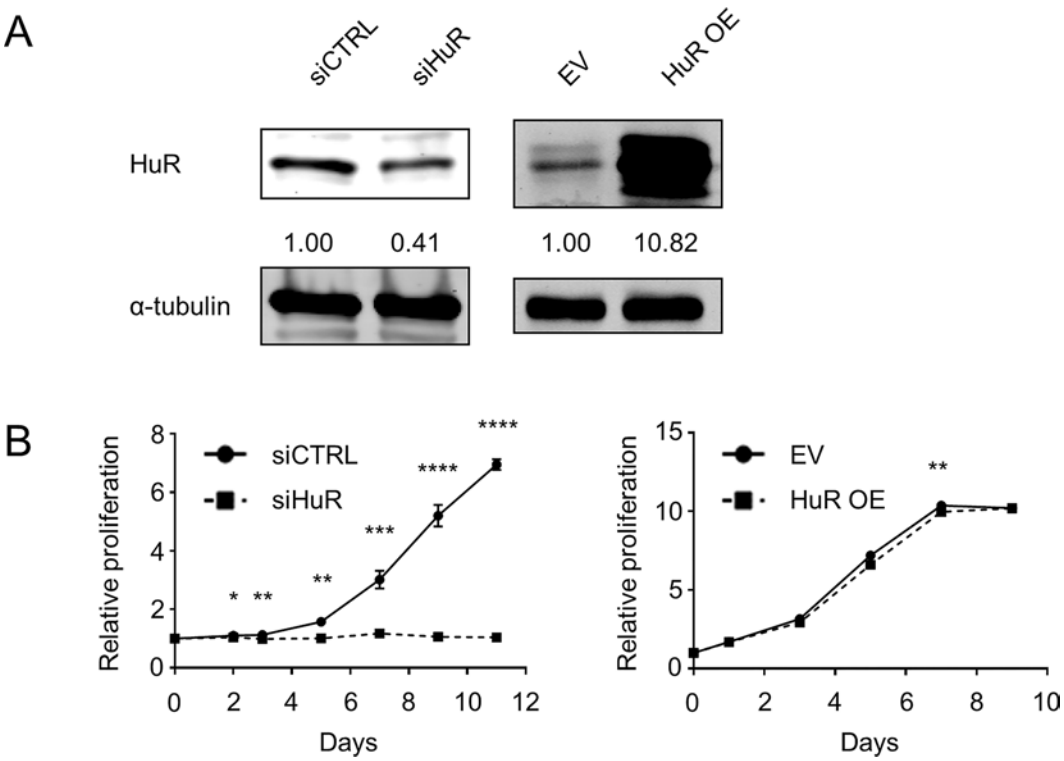

Supplementary Figure S2: Short-term proliferation of transiently transfected PL5 cells. **A.** Representative western blot of HuR protein expression in PL5 cells transiently transfected with control siRNA (siCTRL), HuR siRNA (siHuR), empty vector (EV), or HuR overexpression plasmid (HuR OE) for 3 days. Alpha-tubulin was used as normalization control. **B.** Relative proliferation of transiently transfected PL5 cells, as determined by measurement of dsDNA content by PicoGreen staining. Each data point represents the mean of 5 independent experiments ± SEM. * = $p < 0.05$; ** = $p < 0.01$; *** = $p < 0.001$; **** = $p < 0.0001$.

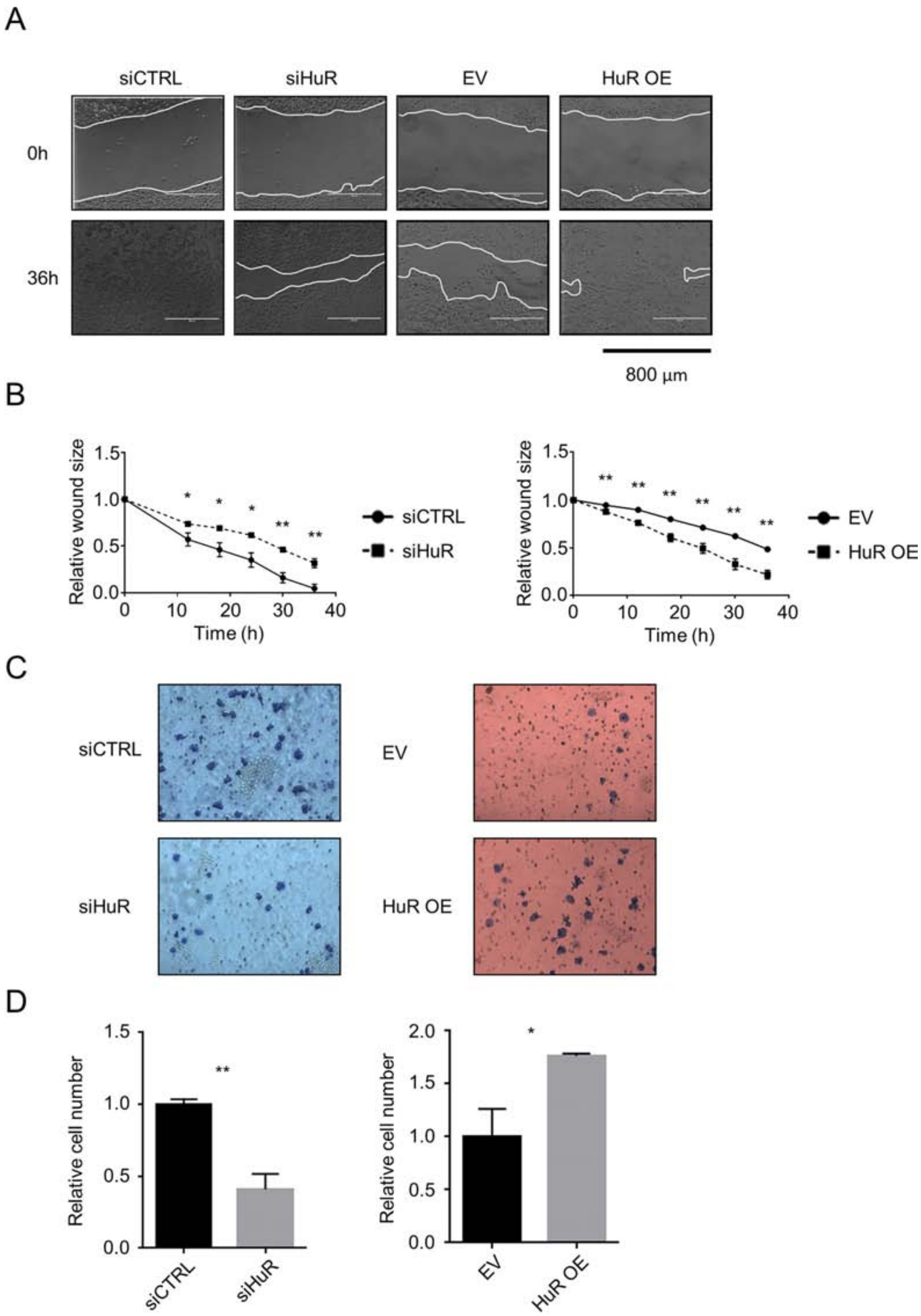

Supplementary Figure S3: Effects of transient HuR silencing and overexpression on PL5 migration and invasion. **A.** Representative images of *in vitro* scratch assays performed with PL5 cells transiently transfected with control siRNA (siCTRL), HuR siRNA (siHuR), empty vector (EV), or HuR overexpression plasmid (HuR OE) for 3 days. Images were taken at 0 h and 36 h post-scratch. Leading edges were outlined in white for ease of visualization. **B.** Quantification of the rate of scratch closure, as measured by change in wound size relative to the 0 h time point. Each data point represents the mean of 3 independent experiments ± SEM. **C.** Representative images of Matrigel invasion assays performed with PL5 cells transiently transfected with siCTRL, siHuR, EV, or HuR OE for 3 days. Cells that invaded through the Matrigel and onto the basal surface of transwell inserts were stained with Differential Quik and photographed at 20X magnification. **D.** Quantification of Matrigel invasion assays. Values were normalized to the number of cells in the control groups. Each bar represents the mean of 3 independent experiments ± SEM. * = $p < 0.05$; ** = $p < 0.01$.

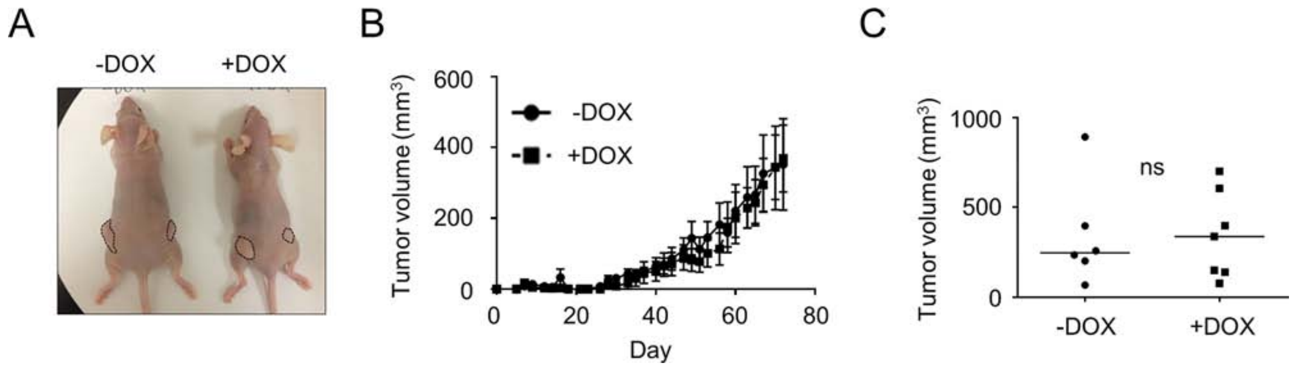

Supplementary Figure S4: Tumor growth of Mia.CTRL xenografts. A. Representative images of subcutaneous Mia.CTRL tumors on the flanks of nude female mice, at the termination of the experiment (day 72). **B.** Tumor growth curves of Mia.CTRL xenografts. Mice were fed normal diet or 200 mg/kg DOX diet starting on day 0 (date of xenograft injection). Each data point represents the mean ± SEM (*n* = 6 for –DOX group, and *n* = 7 for +DOX group). **C.** Plot of all tumor volumes on the final day of the Mia.CTRL xenograft experiment (day 72). Horizontal bars represent the median tumor volumes. *ns* = non-significant.

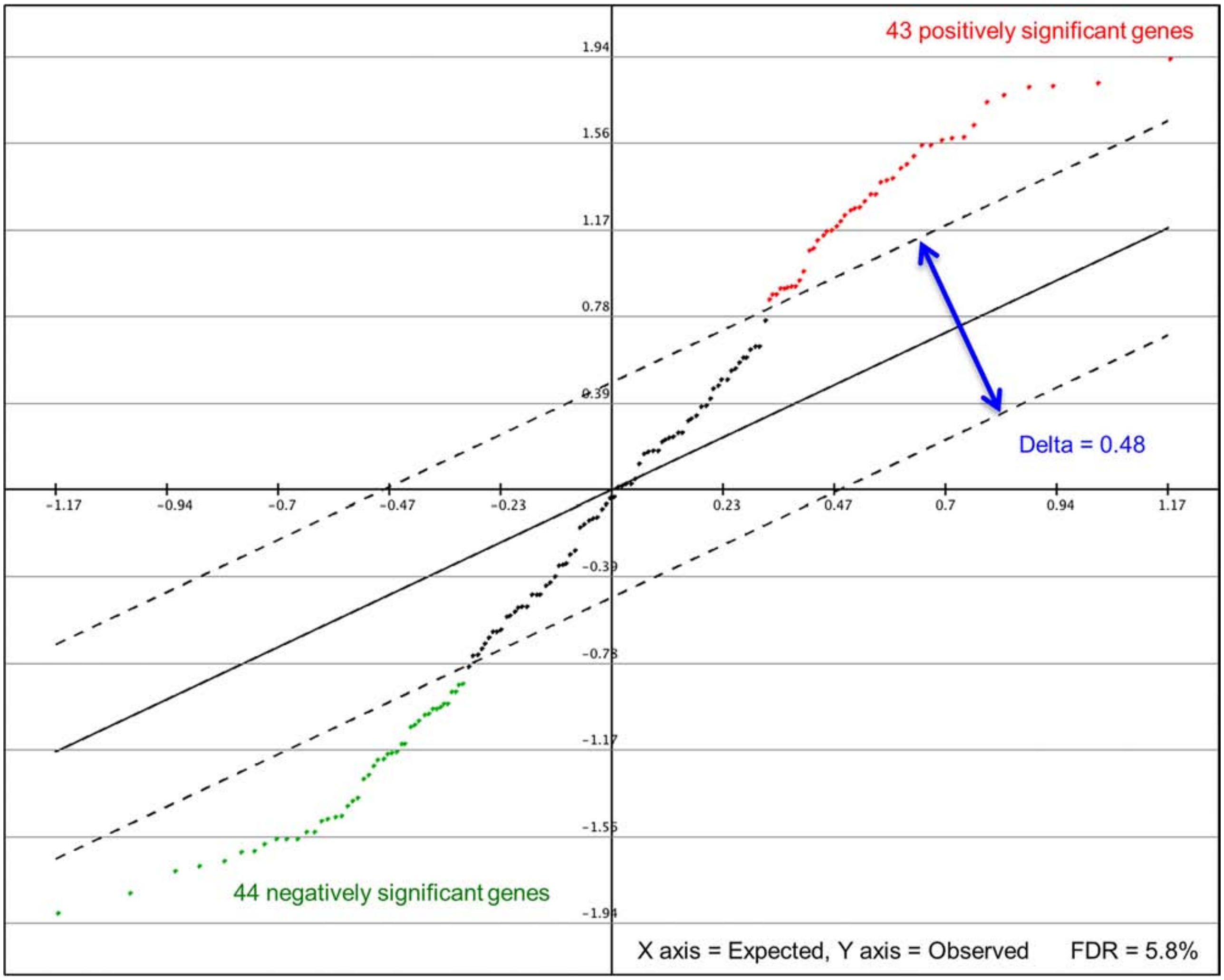

Supplementary Figure S5: Significance analysis of microarrays (SAM). SAM of mRNA transcripts differentially regulated in MIA PaCa-2 cells transfected for 72 hours with HuR siRNA, compared to cells transfected with control siRNA. FDR = false discovery rate.

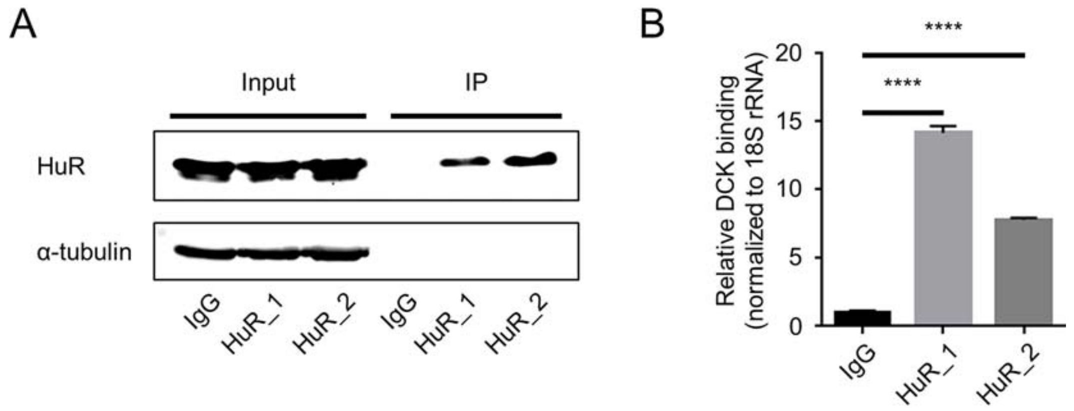

Supplementary Figure S6: mRNP-IP validation. A. Western blot validation of mRNP-IP experiment. **B.** qPCR validation of mRNP-IP experiment. The relative expression of *DCK* mRNA, a previously identified HuR binding target, in the mRNP-IP RNA samples is shown. 18S rRNA was used as normalization control. IP = immunoprecipitate. **** = $p < 0.0001$.

Supplementary Table S1. Complete gene list of NanoString Technologies nCounter® GX Human Cancer Reference Kit.

Supplementary Table S2. HuR-bound transcripts identified by NanoString nCounter® analyses.

Gene	Fold enrichment
SFPQ	1,289.24
BIRC5	1,273.03
TP53	1,068.58
CTNNB1	788.09
CD44	758.39
PRKAR1A	606.66
MYC	285.82
PCTK1	238.12
ITGB1	194.52
DEK	176.90
CASP2	173.47
TOP1	169.16
TFDP1	164.53
ETV6	147.55
MTA1	137.25
TUBB	134.86
YES1	128.01
PTEN	122.45
PGK1	120.63
GAPDH	106.52
HIF1A	103.17
PTPN11	87.13
CSK	80.97
KRAS	77.88
PDGFA	73.38
HPRT1	72.85
NQO1	70.36
YY1	68.76
HSP90AB1	67.34
CCND1	66.65
STAT3	60.78
RB1	59.83
S100A4	44.72
NRAS	34.04
NF1	31.62

Full list of transcripts that were enriched in MIA PaCa-2 HuR mRNP-IP samples, compared to control IgG mRNP-IP samples. Fold enrichment = average of the transcript expression in HuR mRNP-IP samples vs. average of the transcript expression in IgG mRNP-IP samples.

Supplementary Table S3. Biological processes enriched in transcripts regulated by HuR. Lists of biological process gene ontology (GO) terms significantly enriched in the HuR-regulated transcripts, as analyzed by DAVID. Positive = GO terms significantly enriched in the list of transcripts upregulated upon HuR silencing (see Table 1, *left*). Negative = GO terms significantly enriched in the list of transcripts downregulated upon HuR silencing (see Table 1, *right*). Binders = GO terms significantly enriched in the list of transcripts that are direct HuR targets (see Table S2). Count = number of genes associated with the GO term. Fold enrichment = fold enrichment of the GO term in the associated genes, compared to the background. FDR = false discovery rate.
